# Whole-body recruitment of glycocalyx volume during intravenous adenosine infusion

**DOI:** 10.1002/phy2.102

**Published:** 2013-10-11

**Authors:** Judith Brands, Judith van Haare, Hans Vink, Jurgen W G E VanTeeffelen

**Affiliations:** 1 Department of Physiology, Cardiovascular Research Institute Maastricht (CARIM), Maastricht UniversityMaastricht, the Netherlands; 2 Department of Medicine, Cardiovascular Institute, School of Medicine, University of PittsburghPittsburgh, Pennsylvania

**Keywords:** Adenosine, glycocalyx, indicator dilution, systemic

## Abstract

Adenosine-mediated recruitment of microvascular volume in heart and muscle has been suggested to include, in addition to vasodilation of resistance vessels, an increased accessibility of the endothelial glycocalyx for flowing plasma as a result of an impairment of its barrier properties. The aim of the current study was to investigate the effect of systemic intravenous administration of adenosine on the glycocalyx-dependent exclusion of circulating blood at a whole-body level. In anesthetized goats (*N* = 6), systemic blood-excluded glycocalyx volume was measured by comparing the intravascular distribution volume of the suggested glycocalyx accessible tracer dextrans with a molecular weight of 40 kDa (Dex-40) to that of circulating plasma, derived from the dilution of labeled red blood cells and large vessel hematocrit. Systemic glycocalyx volume was determined at baseline and during intravenous infusion of adenosine (157 ± 11.6 μg/kg min^−1^). Blood-inaccessible glycocalyx volume decreased from 458.1 ± 95.5 to 18.1 ± 62.2 mL (*P* < 0.01) during adenosine administration. While circulating plasma volume did not change significantly (617.1 ± 48.5 vs. 759.2 ± 47.9 mL, NS), the decrease in blood-excluded glycocalyx volume was associated with a decrease in Dex-40 distribution volume (from 1075.2 ± 71.0 to 777.3 ± 60.0 mL, *P* < 0.01). Intravenous administration of adenosine is associated with a robust impairment of whole-body glycocalyx barrier properties, reflected by a greatly reduced exclusion of circulating blood compared to small dextrans. The observed decrease in Dex-40 distribution volume suggests that the reduction in glycocalyx volume coincides with a reduction in tracer-accessible vascular volume.

## Introduction

Maximal coronary hyperemia, required for the measurement of fractional flow reserve and coronary flow reserve, is clinically achieved by administration of the vasodilator adenosine, either by continuous intravenous infusion or as an intracoronary bolus (Wilson et al. 1990; Jeremias et al. 2000; Casella et al. 2004). Recent studies indicate that during adenosine administration, besides vasodilation of the resistance vessels, also the barrier properties of the endothelial glycocalyx may change, allowing an increased accessibility for circulating blood (Klitzman and Duling 1979; Desjardins and Duling 1990; Platts and Duling 2004; VanTeeffelen et al. 2005; Brands et al. 2010). Recently, we demonstrated in anesthetized goats that maximal coronary blood volume following intracoronary administration of adenosine was almost identical with and without prior glycocalyx degradation by the enzyme hyaluronidase, indicating that adenosine allows almost full access of circulating blood into the glycocalyx in the coronary circulation (Brands et al. 2010). This raises the question whether systemic infusion of adenosine would have the same potency to decrease the barrier properties of the glycocalyx throughout the circulation and, if so, how the cardiovascular system would cope with the relatively large additional intravascular space which becomes accessible to the blood; The entire glycocalyx volume in the body has been estimated to be 20–25 mL/kg body weight (Nieuwdorp et al. 2006a,b) and plain recruitment of this volume for blood perfusion during adenosine would, therefore, confront the cardiovascular system with a severe filling problem.

In the current study, we investigated the effect of intravenous adenosine infusion on whole-body blood exclusion by the glycocalyx. We hypothesized that systemic adenosine infusion decreases blood exclusion by the glycocalyx throughout the vasculature, and that the anticipated fall in peripheral vascular resistance is counteracted by a reduction in total microvascular volume, in a similar manner as was previously reported during provoked perturbation of the glycocalyx by intravenous infusion of bacterial lipopolysaccharide (LPS) and glucose in humans (Nieuwdorp et al. 2006b, 2009).

In anesthetized goats, systemic blood-excluded glycocalyx volume was determined from the difference in distribution volume of circulating plasma, derived from the dilution of labeled red blood cells and large vessel hematocrit, and the distribution volume of a tracer for both plasma and glycocalyx volume, dextrans with a molecular weight of 40 kDa (Dex-40) (Nieuwdorp et al. 2006a,b; van Teeffelen et al. 2013). Tracers were infused during control conditions and during intravenous infusion of a dose of adenosine (∼160 μg/kg min^−1^), which is clinically used to evoke maximal coronary hyperemia. Systemic blood pressure, heart rate (HR), and coronary blood flow were measured as well.

## Material and Methods

### Animal preparation

All of the procedures and protocols were approved by the Animal Care and Use committee of the Academic Medical Center, University of Amsterdam. Studies were conducted in accordance with the National Institutes of Health Guide for the Care and Use of Laboratory Animals. Experiments were performed on adult female goats of 17–29 kg (*N* = 6). At the beginning of an experiment, the goats were anesthetized with an intramuscular injection of Nimatek (15 mg/kg, Eurovet Animal Health BV, Bladel, the Netherlands) and Dormicum (0.75 mg/kg, Roche, Basel, Switzerland). Goats were intubated and ventilated with a 1:2 O_2_:air mixture. Anesthesia was maintained by intravenous administration of Sufenta (9.375 μg/kg h^−1^, Janssen-Cilag, Beerse, Belgium), Dormicum (0.625 mg/kg h^−1^, Roche, Basel, Switzerland), and Propofol (10 mg/kg h^−1^, B.Braun, Melsungen, Germany). Depth of anesthesia was adjusted according to stability of femoral artery blood pressure (P_fem_) and HR. Arterial and coronary venous blood gases, arterial hematocrit, and pH were measured every 30 min and analyzed using a Radiometer ABL 510 (Radiometer, Copenhagen, Denmark). When necessary, ventilation was adjusted to maintain oxygen and CO_2_ pressures within physiological limits, and sodium bicarbonate administered to avoid acidosis (Brands et al. 2010).

### Surgery

The following surgical procedures were performed. First, catheters were placed in the femoral vein, for the infusion of tracers and adenosine, and via the left carotid artery in the aorta, for arterial blood sampling. Next, a left thoracotomy was performed in the fourth intercostal space and one of the ribs was removed. The great cardiac vein was cannulated via the azygos vein to obtain coronary venous blood samples. A flowprobe (3 mm Transonic flowprobe; Transonic Systems Inc, Ithaca, NY) was placed around one of the major coronary branches (left anterior descending or left circumflex artery) to measure coronary blood flow (*Q*_cor_). The *P*_fem_, *Q*_cor_, and HR (determined from P_fem_) were stored for offline analysis (100 Hz PowerLab Data Acquisition Systems; ADInstruments, Dunedin, New Zealand). At the end of the experimental procedures, a battery was placed on the heart to induce ventricular fibrillation.

### Experimental protocol

After surgery, the preparation was allowed to equilibrate for 30 min. Systemic glycocalyx volume and circulating blood volume were measured first at baseline and subsequently during 20 min of intravenous infusion of 157 ± 11.6 μg/kg min^−1^ adenosine (Wilson et al. 1990; Casella et al. 2004; Kaufmann et al. 2004; Tansley et al. 2004; Park et al. 2006). Adenosine administration was limited to 20 min in total to resemble a clinical dose as close as possible yet long enough to collect a sufficient amount of samples for the measurement of Dex-40 and blood distribution volume. A 5-min delay time was allowed between the start of adenosine administration and the measurement of systemic distribution volumes. Within this time period a new steady state for coronary blood flow, HR, and blood pressure was obtained.

#### Tracers

Systemic glycocalyx volume was determined from the difference in distribution volume of circulating plasma (derived from red blood cell volume and aorta hematocrit) and Dex-40 (100 mg/mL Rheomacrodex; NPBI International, Emmer Compascuum, Netherlands) (Nieuwdorp et al. 2006a,b; van Teeffelen et al. 2013). At the start of the surgery, 40 mL of blood was taken per measurement and centrifuged at 1200 g for 5 min. Subsequently, the centrifuged red blood cells were mixed with sodium fluorescein (250 mg/mL) for 10 min. After being washed, the labeled red blood cells were resuspended in Dex-40 (100 mL).

#### Injection

Previous to each measurement blood was collected (presample), after which the tracers were administered in the femoral vein with a syringe pump (30 mL/min, B.Braun). Before the first injection of the tracers, a single bolus of 5 mL dextrans with a molecular weight of 1 kDa (Promiten; NPBI International) was injected to attenuate the risk for anaphylactic reactions (Zinderman et al. 2006). The tracers were infused within 15 min after the administration of Promiten (Ljungstrom 2007).

#### Sampling

Blood was sampled from both the great cardiac vein and aorta at *t* = 3, 5, 8, and 12 min after the infusion of tracers was stopped. The first sample was taken after ∼3 min to guarantee complete mixture of the tracers with the blood. To collect only blood samples during the administration of adenosine, the last sample, in contrast to the study in human subjects where they sampled up to 30 min (Nieuwdorp et al. 2006a,b), was taken 12 min after the infusion of the tracers.

### Data analysis

Labeled red blood cell fraction was measured using a FACScan analyzer (FACSCalibur; Becton Dickinson, Franklin Lakes, NJ). The fraction of labeled red blood cells was found to be constant between 3 and 12 min after the dextrans were infused (data not shown), and the average value of the data within this period was taken during further analysis. The average fraction of labeled red blood cells versus the total red blood cell pool was used to estimate circulating red blood cell volume (Orth et al. 1998). The circulating plasma volume (V_plasma_) was calculated as:


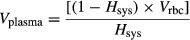


where V_rbc_ is the circulating red blood cell volume and H_sys_ is the hematocrit. Total circulating blood volume was defined as the sum of V_plasma_ and V_rbc_. After measuring the fraction of labeled red blood cells, blood was centrifuged and the plasma collected and stored at −20°C until analyzed.

**Figure 1:**
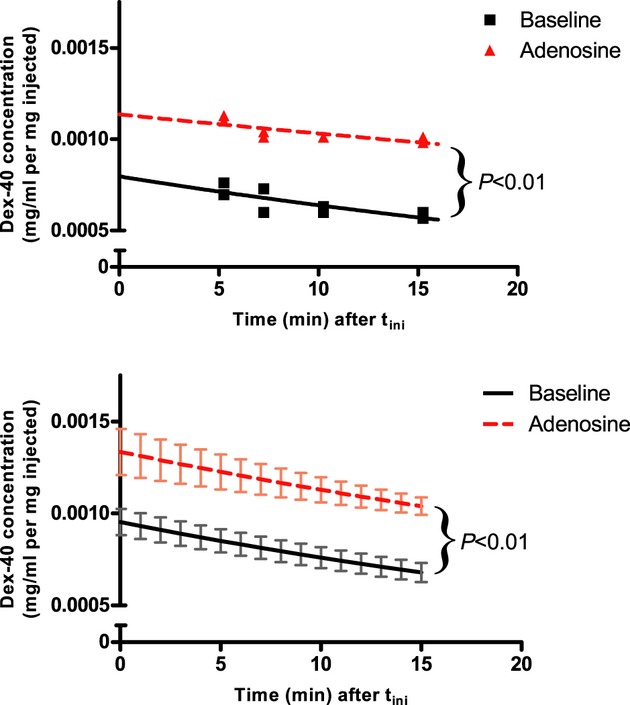
Top: A typical example of measured Dex-40 normalized to the amount of Dex-40 given (mg/mL per mg injected tracer) over time and the exponential fit to determine the concentration at t_ini_ (start of tracer infusion) for baseline (▪) and adenosine (▲). Bottom: Averaged plasma Dex-40 concentration curve normalized to the amount of Dex-40 given (mg/mL per mg injected tracer) over time at baseline (solid line, y = 0.00095e^−0.023t^) and during adenosine (dashed line, y = 0.0013e^−0.016t^). The averaged concentration curve is calculated using inter- and extrapolation of the measured Dex-40 data at *t* = 3, 5, 8, and 12 min after infusion of tracer using an mono-exponential fit. The standard error of the mean is indicated by the error bars. The clearance of Dex-40 (power of the exponential fit) from the plasma was not statistically different in both measurements. Data are means ± SEM. *P* < 0.01, difference in concentration of Dex-40 measured between adenosine and baseline measurements at all points in time.

The Dex-40 concentration was calculated by measuring the increase in plasma glucose concentration in the postinfusion samples after hydrolysis of the dextrans (van Kreel et al. 1998). All measured glucose concentrations were corrected for the background glucose level (0.7 ± 0.04 mg/mL) in the blood, measured in the presample. The samples taken from the great cardiac vein and aorta were taken as duplicate measurements. To determine the initial Dex-40 distribution volume, the concentration of Dex-40 at t_ini_ (onset of the infusion of the tracers) was estimated by exponential fitting of the measured Dex-40 concentrations (Nieuwdorp et al. 2006a,b; van Teeffelen et al. 2013), see Figure 1. The distribution volume of Dex-40 was calculated by dividing the amount of dextran given by the background corrected concentration of dextrans at t_ini_ (mg/mL). The clearance rate of the Dex-40 tracer was reflected by the power of the exponential fit (Nieuwdorp et al. 2006a,b).

All results are expressed as means ± SEM. Differences in blood pressure, coronary blood flow, and HR, as well as effects on volumes, hematocrit, initial Dex-40 concentrations, and clearance rate were tested using a *t*-test. A probability value of *P* < 0.05 was considered significant.

## Results

**Figure 2:**
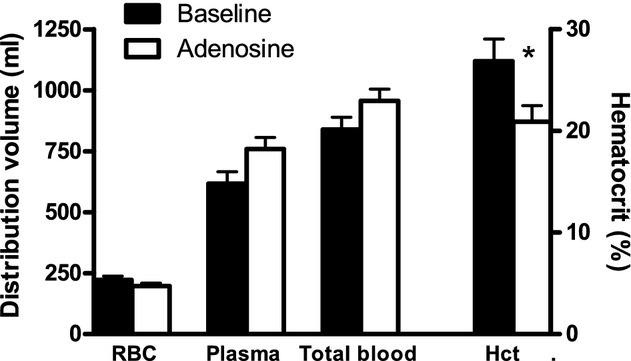
Red blood cell (RBC), plasma and total blood volume, and hematocrit (Hct) at baseline and during adenosine. Data are means ± SEM, *P < 0.05, from baseline hematocrit measurements.

Baseline hemodynamic parameters are presented in Table 1. Comparing baseline with adenosine measurements there was a 3.0 ± 0.5-fold increase in coronary flow (*P* < 0.01), a significant increase in HR and a modest reduction in femoral artery blood pressure (*P* < 0.08). There was also a significant decrease in hematocrit from 26.9 ± 2.2% at baseline to 20.9 ± 1.6% during adenosine, see Figure 2.

**Table 1:** Hemodynamic parameters at baseline and during adenosine (*N* = 6)

	Baseline	Adenosine
P_fem_ (mmHg)	91.0 ± 7.8	72.5 ± 4.9
HR (beats/min)	117.7 ± 7.9	132.3 ± 7.0*
Q_cor_ (mL/min)	47.7 ± 7.6	133.6 ± 19.8*

Values are means ± SEM,

* significant from baseline (*P* < 0.05).

**Figure 3:**
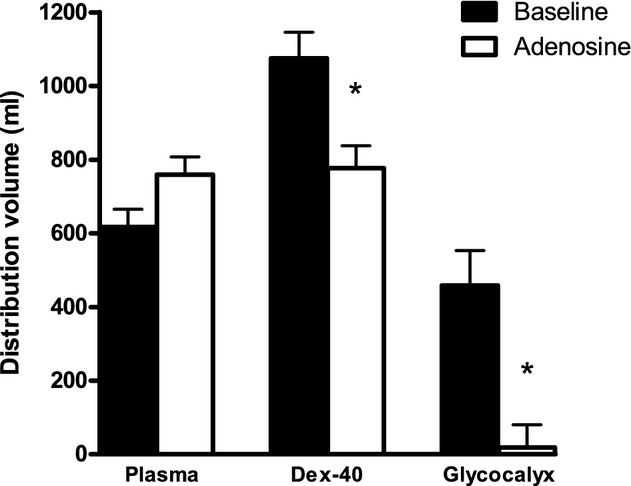
Plasma, Dex-40, and glycocalyx volume at baseline and during adenosine. Data are means ± SEM, *P < 0.05, from baseline volume measurements.

The averaged extrapolated clearance curves of Dex-40 are depicted in Figure 1 (bottom). At baseline the concentration of Dex-40 at t_ini_ was 9.5 × 10^−4^ ±0.7 × 10^−4^ mg/mL per mg infused tracer. This concentration was significantly increased to 13.3 × 10^−4^ ±1.3 × 10^−4^mg/mL per mg infused tracer during adenosine (*P* < 0.01). Consistent with the diminished dilution of dextrans, the Dex-40 distribution volume decreased significantly from 1075.2 ± 71.0 mL at baseline to 777.3 ± 60.0 mL during adenosine (*P* < 0.01). The clearance rate of the Dex-40 from the plasma was the same in both measurements, reflected by an unchanged exponential coefficient (0.023 ± 0.002 min^−1^ at baseline and 0.016 ± 0.003min^−1^ during adenosine, NS). The circulating red blood cell volume, plasma, and total blood volume at baseline were not different from the volumes measured during adenosine administration; volumes are depicted in Figure 2. The difference between the circulating plasma and Dex-40 distribution volume, that is, the blood-inaccessible glycocalyx volume, decreased significantly (*P* < 0.01) comparing baseline with adenosine measurements (from 458.1 ± 95.5 mL to 18.1 ± 62.2 mL, respectively), see Figure 3.

When comparing the different volumes given in Figure 3, it can be seen that at baseline the blood-inaccessible glycocalyx volume and the circulating plasma volume are nearly equal in size, while during adenosine infusion the distribution volume of Dex-40 closely corresponds to that of the circulating plasma volume.

## Discussion

In the present study in anesthetized goats, we found a ∼0.5 L difference in estimated whole-body distribution volume between circulating blood and Dex-40 under control conditions, indicating a substantial exclusion of circulating blood by the glycocalyx. During intravenous adenosine administration, this difference in distribution was almost completely lost. These data demonstrate the potency of adenosine to impair the barrier properties of the glycocalyx throughout the circulation and substantiate our previous study in which the adenosine-induced blood volume increase in the goat coronary circulation was indicated to include substantial recruitment of glycocalyx volume as well (Brands et al. 2010).

### Hemodynamics

Adenosine is commonly used in the clinic as it is a potent vasodilator of the coronary bed. Indeed, we found a threefold increase in coronary flow during intravenous infusion of a dose used in patients, in the face of a ∼20 mmHg decrease in arterial blood pressure (Table 1). The decrease in blood pressure was not significant (*P* < 0.08), unlike the effects reported on anesthetized dogs (Rowe et al. 1962; Crystal et al. 1988; O'neill et al. 1989; Desjardins and Duling 1990). Furthermore, we also observed a significant increase in HR during the adenosine infusion. Divergent effects of adenosine on HR have been reported in anesthetized animals (Rowe et al. 1962; Crystal et al. 1988; O'neill et al. 1989). Crystal et al. (1988) observed a bradycardia during adenosine in their study in dogs, and suggested that this was due to direct suppression of pacemaker activity in the sinoatrial node by adenosine (James 1965) which was adequate to override the baroreflex-mediated increase in HR associated with aortic hypotension. The effect of adenosine on the sinoatrial node was, however, shown to be dose dependent (James 1965), and this seems to well explain the differences reported in literature. Thus, at a low dose, 0.53 mg/kg min^−1^, an increase in HR was reported (Rowe et al. 1962), whereas at a 2.5 times higher dose the HR appeared not to change significantly (O'neill et al. 1989), and only at a dose four times as high the HR was shown to be reduced, such as in the study of Crystal et al. (1988). Based on these results, we suggest that the dose used in our study, 157 ± 11.6 μg/kg min^−1^, enabled the baroreflex-mediated sympathetic increase to dictate the HR response.

Hematocrit at baseline (26.9 ± 2.2%) was lower than the hematocrit measured in awake goats the day before an experiment (32.6 ± 1.6% [Brands et al. 2010]). The lower hematocrit is explained by the sampling of 80 mL of blood for the labeling of red blood cells that was compensated with the infusion of fluid, in combination with the induction of anesthesia and surgery. Furthermore, hematocrit was significantly reduced during adenosine administration, see Figure 2. Most likely, hematocrit is decreased during the experiment because of dilution of the blood by the infusion of Dex-40 (100 mL) as well as Ringer (B.Braun, Melsungen, DE) that was given as infusion fluid for the duration of the entire experiment. A smaller contribution to the observed reduction in hematocrit may also be expected from the release of fluid that was trapped in the glycocalyx at baseline into the circulation during adenosine.

### Effect of adenosine on endothelial glycocalyx

The increase in coronary blood flow during adenosine has traditionally been contributed to a relaxation of predominantly the distal arterioles which have been shown to be most sensitive to adenosine (Kanatsuka et al. 1989; Habazettl et al. 1994). In addition to resistance vessel relaxation, adenosine has also been indicated to increase perfused microvascular volume by modulation of the glycocalyx. First, Klitzman and Duling (1979) observed a three- to fourfold increase in capillary tube hematocrit, using intravital microscopy, when cremaster muscle was superfused with adenosine. They suggested that the presence of a slow-moving plasma layer with a thickness of 1.2 μm, representing the glycocalyx, contributed to the initial low hematocrit in capillaries, and that a change in this layer could explain the robust increase in capillary tube hematocrit. Later, Duling and coworkers showed that there was indeed an apparent decrease in exclusion of large dextrans by the glycocalyx when adenosine was topically applied on the cremaster muscle (Desjardins and Duling 1990; Platts and Duling 2004). These observations were more recently evaluated in the coronary circulation of large animals. In dog hearts, the adenosine-mediated increase in coronary conductance was observed to exceed maximum conductance during coronary reactive hyperemia, but only in the presence of an intact glycocalyx (VanTeeffelen et al. 2005). Degradation of the glycocalyx with the enzyme hyaluronidase revealed an equally increased conductance of reactive hyperemia and adenosine-induced hyperemia in the heart due to an increase in the former without a change in the latter. These data indicate that the microvascular resistance offered by the glycocalyx was already reduced during adenosine-mediated hyperemia, and suggest an increased accessibility of the glycocalyx by circulating blood during adenosine administration in the coronary circulation. More evidence was provided when we showed, using the indicator dilution technique in goat hearts, that the maximal coronary blood volume following administration of adenosine was similar with and without prior hyaluronidase degradation of the glycocalyx, indicating that adenosine and hyaluronidase potentially reduce blood-inaccessible glycocalyx volume in the coronary circulation to a similar extent (Brands et al. 2010).

### Methodological considerations

In the current study, blood-inaccessible glycocalyx volume was defined as the difference between the distribution volume of Dex-40 and circulating plasma volume determined using labeled red blood cells and large vessel hematocrit. The blood-excluding glycocalyx volume measured at baseline in this study, 22.4 ± 5.2 mL/kg bodyweight, matches nicely with the volumes found in humans and mice, 20–25 mL/kg bodyweight (Nieuwdorp et al. 2006a,b van Teeffelen et al. 2013). The methodology used by us was based on initial intravital microscopic observations that red blood cells and macromolecules are excluded from the glycocalyx in a size- and charge-dependent manner while Dex-40 seems not to be hindered by it (Vink and Duling 2000). Recently, we evaluated the size-selective barrier properties of the glycocalyx at the whole-body level in mice, by comparing the systemic distribution of small (40 kDa) dextrans versus that of intermediate (70 kDa) and large (500 kDa) dextrans using tracer dilution, and versus that of circulating plasma as derived from the dilution of fluorescein-labeled red blood cells and large vessel hematocrit (van Teeffelen et al. 2013). While in control animals circulating plasma and large dextrans were found to distribute in a vascular volume that was considerably smaller than that for Dex-40, tracer differences in distribution volume were greatly diminished in hyaluronidase-treated mice. These observations are consistent with intravital microscopic observations of macro- and microvessels showing that the glycocalyx acts as a molecular filter governing the intravascular distribution of plasma solutes.

A major concern has been previously raised regarding the estimation of the initial volume of distribution of Dex-40 due to the rapid clearance of low-molecular-weight fractions from the circulation (Michel and Curry 2009). In our previous study in mice, we, therefore, approximated initial Dex-40 also by linear backward fitting and found indeed a somewhat higher concentration compared to using the mono-exponential fit (van Teeffelen et al. 2013). Nevertheless, the consequence for the approximation of initial Dex-40 volume was small, that is, 3.6% and 7.5% compared to the mono-exponential approach in the control and hyaluronidase-treated mice, respectively. Similarly, the potential initial overestimation of Dex-40 volume due to its rapid initial clearance from the circulation is anticipated to be of small importance in the current study, also as the clearance of the tracer was not different during adenosine compared to baseline. The overestimation of Dex-40 volume is, therefore, anticipated to be similar for our volume estimation at baseline and during adenosine.

The whole-body measurement does not distinguish where the blood-excluding glycocalyx volume is residing in the circulation. The endothelial glycocalyx thickness has been shown to vary between different vessel types, and has been documented to range from 0.2 to 0.9 μm in capillaries (Vink and Duling 1996, 2000; Henry and Duling 1999; Platts et al. 2003; Platts and Duling 2004; Nieuwdorp et al. 2006a, 2008; Rubio-Gayosso et al. 2006; VanTeeffelen et al. 2008), 2 to 3 μm in small arteries with a diameter of ∼150 μm (van Haaren et al. 2003), and 4 to 5 μm in carotid arteries (Megens et al. 2007). These numbers indicate that during baseline conditions the glycocalyx occupies a large part of the anatomic vascular volume, particularly in the microcirculation. We hypothesize that in tissues with increased adenosine-induced blood flow, including the heart and skin (Kassell et al. 1983; Edlund et al. 1990), recruitment of glycocalyx volume causes a robust increase in vascular blood volume, particularly in the capillaries. The simultaneous dilation of resistance vessels (arterioles) primarily accounts for an increase in flow during adenosine infusion. We expect that in tissues with a reduction in flow during adenosine administration, such as adipose tissue, kidney, liver, and stomach (Kassell et al. 1983; Edlund et al. 1990), the increase in microvascular volume due to glycocalyx recruitment is counteracted by vasoconstriction and loss of number of perfused capillaries, resulting in a reduced blood perfused microvascular tissue volume.

### Glyococalyx recruitment is associated with decrease in Dex-40 volume

In the face of an unchanged circulating blood volume, the decrease in glycocalyx exclusion was associated with a decrease in Dex-40 distribution volume to 72 ± 1.6% of baseline. Also in human subjects, Dex-40 volume was observed to be decreased upon perturbation of the glycocalyx, after 6-h hyperglycemia it was reduced to 85% (Nieuwdorp et al. 2006b), and in type I diabetics to 76% and 82% in patients with and without microalbuminuria, respectively (Nieuwdorp et al. 2006a). A reduction in Dex-40 distribution volume might be explained by a true decrease in vascular anatomic volume as well as a decrease in perfused (and hence tracer accessible) vascular volume. Both aspects have been demonstrated at the capillary level in rodents in response to provoked glycocalyx degradation. Thus, van den Berg et al. (2003) showed that hyaluronan degradation in isolated rat hearts resulted in perivascular capillary edema formation which was associated with a decrease in anatomic diameter of the capillaries, whereas Cabrales et al. (2007) demonstrated a decrease in functional capillary density and increase in nonflowing capillaries in the hamster chamber window model after hyaluronidase treatment. We suggest that these reductions in microvascular blood volume also occur in organs where blood flow during systemic adenosine administration is reduced, likely because of sympathetically stimulated vasoconstriction of the resistance vessels. In contrast, in organs with increased blood flow during adenosine, we envision that microvascular blood volume is increased as a result of both vasodilation of resistance vessels and recruitment of glycocalyx volume for perfusion.

### Clinical translation

Although similar measurements as described in the current study have been performed in human subjects to determine glycocalyx damage in patients with diabetes and during hyperglycemia (Nieuwdorp et al. 2006a,b), these tracer-based glycocalyx volume determinations, and how they are affected by adenosine in, for example, patients with coronary artery disease, cannot routinely be performed in a clinical setting. Nevertheless, an alternative method has recently been developed for noninvasive assessment of glycocalyx changes in the human sublingual microcirculation (Vlahu et al. 2012; Martens et al. 2013; Mulders et al. 2013). This novel analysis uses the dynamic range of the red blood cell column width, monitored with a SDF (side-stream-directed dark-field) camera, to determine the position of the outer edge of the perfused microvessel lumen as a reflection of the glycocalyx barrier properties. Unlike the technique used in the current study where the glycocalyx volume in the entire circulation was determined, SDF imaging is performed on a single vascular bed, enabling the effect of vasoactive stimuli, such as adenosine, to be studied per vessel class and size. Using this approach, it was recently observed that first-degree relatives of patients with premature coronary artery disease were characterized by reduced glycocalyx barrier properties compared to healthy controls, independent of other risk factors (Mulders et al. 2013). It is anticipated, therefore, that evaluation of sublingual glycocalyx damage might be useful for early risk prediction of coronary microvascular disease in patients with symptoms of chest pain. During the routinely applied adenosine infusion to determine coronary flow reserve in these patients, the sublingual microvasculature can be monitored simultaneously to determine glycocalyx recruitment by adenosine.

## Conclusion

In the current study in anesthetized goats, we demonstrate that intravenous administration of a clinical dose of adenosine greatly decreases blood-excluded intravascular glycocalyx volume at whole-body level. During adenosine infusion, the difference between the glycocalyx inaccessible and accessible tracer reduced to nearly zero, illustrating adenosine's potency to robustly increase glycocalyx accessibility for flowing blood. The decrease in blood-inaccessible glycocalyx volume was associated with an almost equivalent decrease in perfused anatomic vascular volume, reflecting the body's compensation to limit the fall in peripheral resistance during adenosine.
